# High Seroprevalence of Jamestown Canyon Virus among Deer and Humans, Nova Scotia, Canada

**DOI:** 10.3201/eid2401.170484

**Published:** 2018-01

**Authors:** Glenn Patriquin, Michael Drebot, Teri Cole, Robbin Lindsay, Emily Schleihauf, B. Lynn Johnston, Kristina Dimitrova, Maya Traykova-Andonova, Angela Mask, David Haldane, Todd F. Hatchette

**Affiliations:** Nova Scotia Health Authority, Halifax, Nova Scotia, Canada (G. Patriquin, B.L. Johnston, D. Haldane, T.F. Hatchette);; Dalhousie University, Halifax (G. Patriquin, B.L. Johnston, D. Haldane, T.F. Hatchette);; Public Health Agency of Canada, Winnipeg, Manitoba, Canada (M. Drebot, R. Lindsay, K. Dimitrova, M. Traykova-Andonova);; Nova Scotia Department of Health and Wellness, Halifax (T. Cole);; Public Health Agency of Canada, Ottawa, Ontario, Canada (E. Schleihauf, A. Mask).

**Keywords:** Jamestown Canyon virus, arbovirus, bunyavirus, California serogroup, *Bunyaviridae*, viral encephalitis, viral meningitis, zoonotic, seroprevalence, Atlantic, Nova Scotia, Canada, viruses

## Abstract

Using residual serum samples from Nova Scotia, Canada, we found that 87.8% of tested deer and an estimated 20.6% of the human population were infected with Jamestown Canyon virus. Human seropositivity reached 48.2% in 1 region. This virus may be an underrecognized cause of disease in Nova Scotia.

Jamestown Canyon virus (JCV), a mosquitoborne arbovirus, belongs to the California serogroup (CSG) in the *Bunyaviridae* family. The primary reservoir host for JCV is considered to be the white-tailed deer, although JCV antibodies have been observed in other mammals, including horses, sheep, and cattle (*1*). JCV-associated illness among humans is rarely documented; rates of ≤0.01 cases/100,000 population have been reported in the United States (*2**,**3*). Infections can be asymptomatic or associated with a variety of manifestations, including fever, headache, myalgia, weakness, and seizure (*4**–**6*).

Evidence of JCV in Atlantic Canada was reported in 1981 when 6 of 289 hunter-killed moose during the 1977–1978 hunting season in Nova Scotia (NS) were seropositive (*7*). There is no surveillance for JCV in NS, nor is it on the list of diseases notifiable to Provincial Public Health as defined in the NS Health Protection Act (*8*), but it could be reported as an unusual disease occurance or a disease occuring more frequently than expected. We undertook this study to determine the seroprevalence of JCV in humans and white-tailed deer in this province.

## The Study

During October 30–November 4, 2009, Public Health Agency of Canada (PHAC) staff or private contractors collected blood from the chest or organs of hunted deer in 2 check stations in NS: Italy Cross (community A) and Lunenburg (community B) (Figure). Hunters visiting a check station collected approximately 10% of all samples analyzed. Blood samples were collected from 82 deer (40 from community A, 42 from community B). All except 3 of the deer were harvested in zone 102 (https://novascotia.ca/natr/draws/deerdraw/ddZones.asp#zone102) in southern NS, which includes community A and community B.

**Figure:**
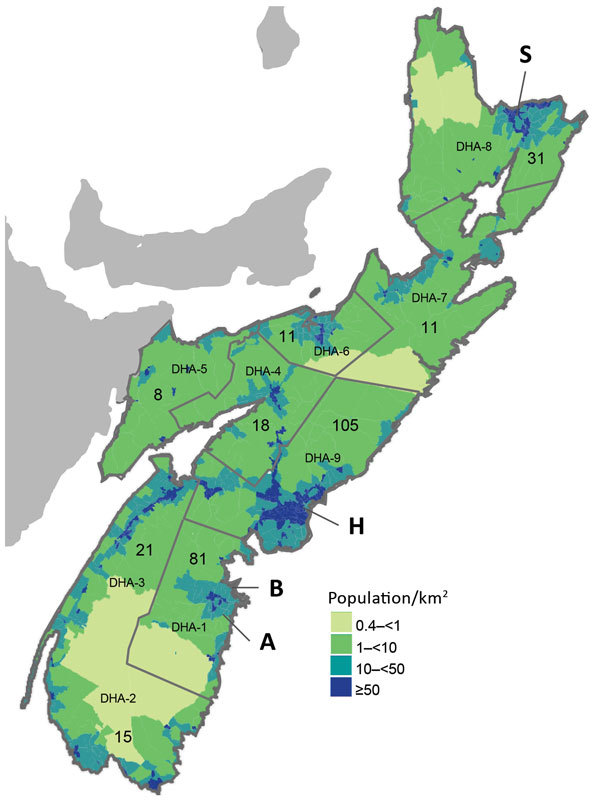
Population density and boundaries of DHAs in Nova Scotia, Canada, at the time of sample collection from white-tailed deer in 2009 and humans in 2012 for study of Jamestown Canyon virus seroprevalence. H, capital city of Halifax; S, Sydney; A, community A; B, community B. Numbers on map indicate number of human serum samples screened in each DHA. Population density map modified from its original format (*9*). DHA, District Health Authority.

To evaluate prevalence of JCV among humans, we used residual human serum specimens submitted for prenatal screening and testing for cholesterol, electrolytes, or HIV during May 1–August 30, 2012, that had been used in a previous serosurvey (*10*). We calculated sample size based on estimated seroprevalence of 20% among the NS population, with precision of ±5% and the ability to detect a statistically significant difference (p = 0.05) between District Health Authority (DHA) 1, DHA 9, and the remaining DHAs (Figure). Because of high seropositivity among hunter-killed deer in DHA 1, humans were oversampled in DHA 1 to facilitate comparisons with other DHAs. We based human population estimates for 2012 on census data from Statistics Canada. We used Stata version 13.0 (StataCorp, College Station, TX, USA) for statistical analysis and used the Stata *svy* prefix command to incorporate sampling weights to produce estimates representative of the NS population. We stratified serum samples by age, sex, and DHA, with sampling proportionate to the NS population in 10-year age groups for those 10–59 years of age and a 5-year age group for those 60–64 years of age. We used Pearson χ^2^ and corrected χ^2^ statistics to test for significant differences based on DHA and sex, respectively. We used logistic regression to test for a significant increasing trend in seroprevalence by age group.

The Research Ethics Board of each DHA approved the serosurvey; 1 board required an opt-out option by publicizing the study and asking patients to self-identify nonparticipation at the time of collection. No patient declined participation.

We shipped the samples to the National Microbiology Laboratory, where serum samples were stored at −80°C. JCV antibodies in human and deer specimens were determined by using an arbovirus plaque-reduction neutralization assay (PRNT); a titer of >1:20 was considered positive (*10*). Snowshoe hare virus (SSHV) or JCV and various dilutions of deer serum samples were incubated at 37°C for 1 h in tissue culture media, then added to Vero cell monolayers. After 1 hour of incubation at 37°C, an agar overlay was added; the plates were incubated in CO_2_ for 3 days. An overlay containing neutral red was added to visualize plaque formation. Serum samples that inhibited >90% of plaque formation relative to virus controls, the highest positive serum dilution of the titration endpoint, were considered positive for viral antibodies. PRNT results were considered positive if the neutralizing antibody titer was >20; additional PRNT endpoint titrations were used to discriminate between related California serogroup viruses. Serum samples that demonstrated a 4-fold or greater difference in PRNT titers between SSHV and JCV were used to identify the virus to which the individual was previously exposed. JCV and SSHV strains used in PRNT assays were NY74-31 and SK75-93, respectively.

JCV antibody titers in samples from 72 of 82 deer ranged from 1:20 to >1:1,280; overall seroprevalence was 87.8% (95% CI 78.7%–94.0%; Table 1). Measured by using the Fisher exact test, seropositivity did not differ significantly based on sampling location, and JCV exposure did not differ by sex: 87.5% (35/40) of male deer and 91.9% (34/37) of female deer were seropositive (Table 1). Seropositivity increased with age (75.0% of immature deer and 93.1% of adult deer were JCV-positive), but the difference was not statistically significant (p = 0.0567).

**Table 1:** White-tailed deer seropositivity for Jamestown Canyon virus, Nova Scotia, Canada*

No. white-tailed deer	No. (%) seropositive	No. seronegative	p value
Total, n =82	72 (87.8)	10	NA*
Community			1.000
A, n =40	35 (87.5)	5	
B, n =42	37 (88.1)	5	
Sex			0.713
Male, n =40	35 (87.5)	5	
Female, n =37	34 (91.9)	3	
Unknown, n =5	3 (60.0)	2	
Age			0.0567
Adult, n =58	54 (93.1)	4	
Immature, n =24	18 (75.0)	6	
*NA, not applicable.			

Of 301 human serum specimens tested for JCV antibodies, 81 were positive (Table 2). After adjusting for survey design, the estimated overall seroprevalence for JCV in NS was 20.6% (95% CI 16.0%–25.9%). The DHA 1 seropositivity of 48.2% (95% CI 37.2%–59.1%) was significantly higher than those of DHAs 2–8 (combined seroprevalence: 22.6%, 95% CI 15.8%–31.4%) and that of DHA 9 (seroprevalence 15.2%, 95% CI 9.5%–23.5% [Table 2]). Estimated seroprevalence was higher for men and boys than for women and girls (26.8% vs. 14.4%, respectively; p = 0.013) and increased with age (p = 0.024): 10.8% were seropositive among those 10–19 years of age, and 33% were seropositive among those 60–64 years of age (Table 2).

**Table 2:** Human Jamestown Canyon virus seropositivity by sex and age, Nova Scotia, Canada

Characteristic	No. tested	No. (%) positive	Adjusted proportion (95% CI)	p value*
Sex				0.013
M	151	51 (33.8)	26.8 (19.9–35.0)	
F	150	30 (20.0)	14.4 (9.3–21.5)	
Age group, y				0.024
10–19	43	6 (14.0)	10.8 (4.0–25.8)	
20–29	54	13 (24.1)	20.4 (11.0–34.5)	
30–39	52	12 (23.1)	14.1 (6.9–26.7)	
40–49	60	15 (25.0)	20.4 (1.6–33.3)	
50–59	61	21 (34.4)	27.8 (17.1–41.7)	
60–64	31	14 (45.2)	33.0 (17.7–53.0)	
District Health Authority				0.004
1	81	39 (48.2)	48.2† (37.4–59.1)	
2–8	115	26 (22.6)	22.6† (15.8–31.4)	
9	105	16 (15.2)	15.2† (9.5–23.5)	
Total no.	301	81 (26.9)	20.6 (16.0–25.9)	

*Pearson χ^2^ for test by DHA; corrected Pearson χ^2^ for test by sex; logistic regression test for trend by age group. †Adjustment was unnecessary for estimates made by the DHA because weighting was done by this authority.

## Conclusions

In this study, we found that >20% of the tested residual serum samples of persons from Nova Scotia had antibody evidence of infection with JCV. The highest seroprevalence was 48% in specimens tested from 1 of the 9 DHAs. JCV antibodies were found in 88% of hunter-killed deer, higher than in studies in the United States (*12*,*13*).

A seroprevalence rate of 20.6% in persons living in NS is comparable to that seen in reports of other locations (*14*,*15*). The lowest rates of JCV in NS are in DHAs in which the province’s 2 most populous cities, Halifax (DHA 9) and Sydney (DHA 8), are located. In addition to correlating JCV seropositivity with geographic location, this study demonstrated associations of seropositivity with male sex and increasing age in both sexes. These findings support prior data that identified increasing age as predictive for JCV infection, reporting an approximate 10-fold increase in JCV antibodies among persons in Alaska when comparing those <4 years of age with those >65 years of age (*15*).

A limitation of this study is the relatively small number of serum samples, which may not represent the population at risk for this zoonotic disease. The use of anonymized residual serum samples did not enable us to collect data on outdoor activities, exposure history, or travel, so we cannot rule out the possibility that exposure to JCV occurred outside the respective DHA.

We provide serologic evidence of JCV infection in humans and deer in NS and suggest that central nervous system infections (e.g., viral encephalitis and viral meningitis) caused by JCV have been unrecognized. Until more is known of JCV’s clinical effect in this province, evidence of its circulation should prompt consideration as a possible etiology of aseptic central nervous system disease.
